# Effort-Reward-Imbalance, Burnout, and Depression Among Psychiatrists 2006 and 2016-Changes After a Legislative Intervention

**DOI:** 10.3389/fpsyt.2021.641912

**Published:** 2021-04-06

**Authors:** Petra Beschoner, Jörn von Wietersheim, Marc N. Jarczok, Maxi Braun, Carlos Schönfeldt-Lecuona, Roberto Viviani, Lucia Jerg-Bretzke, Maximilian Kempf, Aniela Brück

**Affiliations:** ^1^Department of Psychosomatic Medicine and Psychotherapy, Ulm University Medical Center, Ulm, Germany; ^2^Clinic for Ophthalmology, Marienhospital Osnabrück, Osnabrück, Germany; ^3^Department of Psychiatry and Psychotherapy III, Ulm University Medical Center, Ulm, Germany; ^4^Institute of Psychology, University of Innsbruck, Innsbruck, Austria

**Keywords:** burnout, depression, effort-reward-imbalance, psychiatrists, job strain

## Abstract

**Background:** Physicians, especially psychiatrists, have a high risk of job-related stress, and mental impairment. In our study we examined changes in private and occupational stress factors and mental health within a decade. The legislative reduction of physicians' working hours in Germany during this period made it possible to investigate the impact of working hours in particular.

**Methods:** Questionnaires were administered at two psychiatrist meetings (2006 and 2016) about job and family situation, depressiveness, burnout and effort-reward imbalance. A total of *N* = 1,797 datasets were analyzed.

**Results:** Working hours and free weekends were associated with mental health indices. Correlation analyses showed that a reduction in weekly working hours and working days at weekends was related to reduced scores for effort-reward-imbalance, burnout and depression.

**Conclusions:** Our data show changes in workplace stress and mental health in psychiatrists in a decade in which a reduction in working hours has been required by law. These results can provide indications of effective prevention strategies in the professional context of physicians working in psychiatry.

## Introduction

International studies clearly demonstrate that physicians are at an elevated risk to experience job-related stress and the possible concerns for mental well-being (1–3). The frequency of suicides among physicians is reported to be 1.4–2.7 times higher than in the general population and also higher than in other academic professions, such as chemists, police officers, veterinarians or social workers (4, 5). At the same time, systematic reviews show a significant impact of physicians' own health on the quality of patient care (6, 7). Physicians at acute risk for burnout and depression have an increased malpractice rate (i.e., a higher medical error rate) (6–8).

This highlights the importance of investigating job-related stress and possible influencing factors in the medical profession in order to be able to take preventive action. One important factor modulating the effect of work stress of physicians is long working hours (6, 9). Long working hours and frequent on-call duties lead to high burnout rates (6). Conversely, the reduction of working hours leads to decreased burnout rates (10), as demonstrated in longitudinal studies (11, 12).

Besides burnout rates, a reduction in working hours is also associated with improvements in important mental health indices. Both working time effects are reflected in the effort-reward-imbalance model on job-related stress and its effects on mental health (13, 14). This model represents the theoretical framework based on which we may predict that improvements in working conditions will positively affect burnout and affective well-being indices. Numerous cross-sectional and longitudinal studies among health care professionals clearly demonstrate the association between effort-reward-imbalance and mental health (9, 13, 15). The effect of working hours in psychiatrists is of particular interest since the suicide rate for physicians working in the psychiatric field is higher than in most other medical disciplines (4, 16), suggesting that the level of personal and emotional demands is nowhere higher than among mental health professionals (17–19).

Studies among health care professionals originate mostly from outside Europe and/ or comprise of relatively small (*N* < 800) cohorts. However, the legislative and health care systems are somewhat different between Europe and the rest of the world. In 2006, a nationwide survey of hospital physicians' working hours in Germany showed that the legal regulations differed widely from those of other European countries and that the workload of physicians in Germany in particular was very high by international standards. One reason was that the regulation of on-call duties in Germany led to longer working hours compared to international standards (20). In a survey of the participants of the annual meeting of the German psychiatric association (deutsche Gesellschaft für Psychiatrie, Psychotherapie, Psychosomatik und Nervenheilkunde DGPPN) conducted in 2006, we found high rates of occupational stress, burnout and depression when compared to the general population. In 2007 the Working Hours Act came into force in Germany (21). Years earlier, the European Court of Justice had ruled in favor of Spanish hospital physicians in a landmark decision on the earlier dispute as to whether on-call duty is working time or not. German lawmakers then followed suit (22). The Working Hours Act was amended in 2003 to comply with European law. Accordingly, also on-call duty became working time in Germany (22). This meant that on-call duty, where the physician is on-site at the hospital or practice, had to be counted as working time and counted in the legally permissible total working time. Previously, on-site physician on-call time was counted as on-call time that could be performed from home. The legislative change of 2007 thus reduced the total working time allowed for physicians, which, apart from few regulated exceptions, is set to 48 h in Germany (23).

This legislative change offered the opportunity to investigate regulatory effects on working hours and the expected improvements in job-related stress, burnout and depressiveness in psychiatrists. To this end, we undertook a second survey at the same annual meeting, 10 years after the first survey, adopting a similar setting and design to compare mental health indices of congress participants between 2006 and 2016. We expected a reduction of working hours as an effect of the intervention, accompanied by a reduction in job-related stress, burnout, and depression.

The positive effects of reducing working hours (measured as full-time equivalents units) have already been investigated by Shanafelt et al. (12), reporting improvements in burnout and satisfaction. However, this study examined the effects of voluntary reductions (on the worker's own initiative) rather than those following a legislative intervention. However, a reduction in working hours required by law may have a different effect on an employee's reward experience than a self-chosen reduction in working hours by part-time work. We were specifically concerned with the effect of a reduction in working hours initiated by the legislator and implemented by the hospitals on occupational stress in form of the effort-reward-imbalance of psychiatrists.

Because the survey took place 9 years after the legislative change was enacted, there was time for the regulations to be adopted in the health care systems and the possible turbulences due to this legal change to have settled. This extended time period also reduced the risk of bias of short-term effects due to potential uncertainty, anger, frustration or simply the knowledge of pending relief that may have occurred during the transition period of implementation. Furthermore, mental health improvements due to occupational stress may be expected to become manifest some time after a change.

## Methods

We surveyed psychiatrists at the annual meeting of the German Psychiatric Association (DGPPN) in 2006 and 2016. This meeting is the largest of this character and had in 2010 about 8,000 registered participants (24).

The two surveys were designed as cross-sectional studies. In estimating the effect of legislative regulations, we were interested in comparing individuals that had the same position and job description. Over the 10 years that separated the two samples the positions of the individuals in question would likely have changed. For example, junior doctors have become consultants, consultants have retired, and the junior doctors of the later sample were students at the time the first sample was collected. For this reason, we consider a cross-sectional design to be sufficient to capture the information on the effects of legislative change instead of a cohort study following the same participants over a 10 year-period.

Data collection was carried out in the same way for both surveys at the 2006 and 2016 conferences. Paper-and-pencil questionnaires were distributed directly to the congress participants to achieve higher study attendance than expected when surveys are conducted *via* mail or e-mail (25).

The questionnaires included an explanation of the study, a data protection explanation and information on how to give consent to participate. The participants agreed to participate in the study by completing and returning the questionnaires. The sheets were returned anonymously in a locked box at an information desk in the foyer, or by post or e-mail. A contact person at the information stand was available for further questions during the entire survey period. Posters were also used to inform about the survey.

The questionnaires in 2006 and 2016 were almost identical and included questions on demographics such as age, gender and marital status. In addition, questions were asked about the private and professional life situation and the medical history. To assess working hours, participants were required to report the average actual total weekly working hours and the number of weekends without on-call duty. Although working hours may not always be exactly equivalent to workload, we took the former to be a reasonable approximation of the latter in the present study, given the structured character of work in hospitals and in practitioners. Furthermore, indicators such as free week-ends appear to reflect workload accurately.

Standardized instruments were used to record occupational stress (effort-reward-imbalance), burnout and depression (21, 26–30). A total of *N* = 2,426 (in 2006) and *N* = 2,689 questionnaires (in 2016) were enrolled. Of these, *N* = 1,239 (2006) and *N* = 1,088 questionnaires (2016) were returned. Item non-response reduced the available questionnaires to *N* = 997 (2006) and *N* = 800 (2016). Complete information was available from *N* = 997 in 2006 (89.18%) and *N* = 800 in 2016 (90.60%). We compared the samples that were included in the data analysis (analysis sample 2006 and 2016) with the samples with missing information in the rating scales (ERI, BDI-II, MBI) (missing sample 2006 and 2016) with regard to family, occupational and health characteristics using the chi-square test and Mann-Whitney *U*-test, respectively. This allowed us to identify any systematic differences between the analysis samples and the missing samples. This revealed that missingness was completely at random for most predictor variables, with some exceptions. Briefly, excluded participants were on average 4 years older, more often men, not reporting partnership status, less often had children, worked more often in private practice, and more often worked in a senior position. For this reason, while tests of comparisons adjusted for these factors, the mean values taken by these scales as reported in the Results should be taken with caution, given that they may not be fully representative of the sample of congress attendees.

Missing information on age, sex, profession, and outcome variables (ERI, OC, BDI-II, MBI, see below) were excluded (listwise deletion). Missing data on other items were coded with a dummy variable as “not reported,” so they could be retained in the present analysis (see Table 1).

**Table 1:** Comparison of family, occupational and health characteristics.

		**2006**		**2016**		***p***	***V***
		***N***	**%**	***N***	**%**		
**Gender**							
Female		466	46.74	434	54.25	0.002	−0.0746
Male		531	53.26	366	45.75		
**Partnership**							
No		115	11.53	123	15.38	0.046	0.0585
Yes		801	80.34	608	76.00		
Not reported		81	8.12	69	8.62		
**Children**							
No		300	30.09	295	36.88	0.008	0.0736
Yes		682	68.41	497	62.12		
Not reported		15	1.50	8	1.00		
**Full-time contract**							
100%		844	84.65	577	72.12	0.000	0.1569
<100%		149	14.94	210	26.25		
Not reported		4	0.40	13	1.62		
**Main occupation**							
Private practice		310	31.09	225	28.12	0.001	0.0912
Hospital		659	66.10	523	65.38		
Not reported		28	2.81	52	6.50		
**Working position**							
Senior position		568	56.97	461	57.63	0.000	0.0938
Assistance position		401	40.22	287	35.88		
Not reported		28	2.81	52	6.50		
**Acute/chronic illness**							
No		702	70.41	471	58.88	0.000	0.1204
Yes		295	29.59	329	41.12		
Not reported		0	-	-	-		
**Medication intake**							
No		751	75.33	499	62.38	0.000	0.1399
Yes		246	24.67	301	37.62		
Not reported		0	-	-	-		
**Antidepressant intake**							
No		943	94.58	759	94.88	0.784	−0.0065
Yes		54	5.42	41	5.12		
Not reported		0	-	-	-		
**Sedative intake**							
No		969	97.19	775	96.88	0.693	0.0093
Yes		28	2.81	25	3.12		
Not reported		0	-	-	-		
**Analgesic intake**							
No		931	93.38	712	89.00	0.001	0.0778
Yes		66	6.62	88	11.00		
Not reported		0	-	-	-		
**Work missed due to overload**							
No		879	88.16	687	85.88	0.150	0.0340
Yes		118	11.84	113	14.12		
Not reported		0	-	-	-		
**Currently in psychotherapy**							
No		949	95.19	747	93.38	0.098	0.0391
Yes		48	4.81	53	6.62		
Not reported		0	-	-	-		
**Previously in psychotherapy**							
No		706	70.81	555	69.38	0.508	0.0156
Yes		291	29.19	245	30.63		
Not reported		0	-	-	-		
**Depression in medical history**							
No		580	58.17	496	62.00	0.100	−0.0388
Yes		417	41.83	304	38.00		
Not reported		0	-	-	-		
**Of which diagnosed by a specialist**	No	290	67.29	180	58.06	0.010	0.0944
	Yes	141	32.71	130	41.94		
	Not rep.	0	-	-	-		
**Suicide attempt**							
No		973	97.59	782	97.75	0.826	−0.0052
Yes		24	2.41	18	2.25		
Not reported		0	-	-	-		
		**2006**			**2016**			***p***	***R*** **95% Conf. interval**
		***N***	***M***	**SD**	***N***	***M***	**SD**		
Age		997	44.4	8.55	800	48.3	10.4	<0.001	−0.20 (−0.24 to −0.15)
Number of children (when yes)		601	1.69	1.03	481	1.19	1.1	<0.001	0.23 (0.17–0.28)
Work hours (per week)		997	49.3	12	800	46.5	11.9	<0.001	0.11 (0.07–0.16)
	Full-time employees	844	52.1	10.1	577	50.6	9.9	<0.001	0.14 (0.04–0.25)
	Part-time employees	149	33.6	9.6	210	35.3	9.7	0.084	−0.18 (−0.39–0.03)
Free weekends (per month)		997	2.35	0.97	800	2.8	0.965	<0.001	−0.22 (−0.27–0.18)

*P-Values from Chi Square-Test and for continuous data from Mann-Whitney-U-Test. Effect size (31)*.

The institutional review board of the University of Ulm approved the study design (192/16).

### Psychometric Tests

We used the following instruments in our surveys.

#### Beck Depression Inventory

The Beck Depression Inventory II is a self-evaluation tool for assessing the severity of depression. For the survey in 2016, the validated German translation of the BDI-II was used (27). A total of 21 items based on the DSM-IV criteria for major depression are assessed on a four-point scale with increasing severity from 0 to 3 points. Exceptions are the items “changes in sleep habits” and “changes in appetite,” which are assessed on a 7-point scale. As according to the DSM-IV criteria, the items inquire about changes in mood in the previous 2 weeks. With a cut-off of 0–13 points there is evidence of minimal or absent depression, mild depression in 14–19 points, moderate depression in 20–28 points and severe depression in 29–63 points. Inclusion of the relevant DSM-IV criteria in the items provides a convergent validity (32).

For the previous survey in 2006, the validated German version of the original BDI was used (21). The BDI contains 21 items, each with four statements, with increasing severity, for measuring depression symptoms. After summing the points assigned from 0 to 3, the cut-off of a minimal or non-existent symptomatology is set at <11, a mild to moderate symptomatology at 11–17 and a clinically relevant symptomatology at ≥18 points.

As a further development of the BDI some items were added to BDI-II for the complete recording of the DSM-IV criteria for depression, while diagnosis-unspecific items were removed (30). Furthermore, a distinction between the increase and decrease of the items “appetite and sleep” was added (27).

For the comparative analysis of the data from 2006 and 2016, a common BDI categorization was established based on a dichotomization between “depressive” and “non-depressive” status. To categorize participants as “depressive” in the BDI, the cut-off was set at > 10 overall points, while in the BDI-II the cut-off was > 13 overall points (21, 26, 27, 30).

#### Maslach Burnout Inventory D

We used the only German version of the MBI by Maslach and Jackson (MBI-D) (28, 33), which is authorized by Maslach (34). Since the 1992 version of this translated instrument has been revised and reviewed but not yet published, the approval was given by Prof. J. Glaser of the University of Innsbruck.

The MBI-D consists of 21 items ranging from 1 (never) to 6 (very often). The scale contains the three dimensions of burnout: emotional exhaustion (MBI-EE) (9 items), depersonalization (MBI-DP) (5 items) and personal accomplishment (MBI-PA) (7 items). Emotional exhaustion refers to an emotional overload caused by work. Depersonalization means a detached, pejorative, cynical attitude toward the patient. Personal accomplishment reflects feelings of efficiency and fulfillment through the profession. In contrast to the other two dimensions, personal accomplishment is a positive aspect. In the evaluation, higher values for emotional exhaustion and depersonalization therefore indicate a higher risk of burnout, while lower values for personal accomplishment indicate a higher risk of burnout.

The comparison of burnout measured with MBI with other constructs such as stress measured with ERI is possible using the semi-continuous outcome variables (item mean and sum score) of the MBI. We used item means to address the problem of missing items. We also calculated the sum scores, as they allowed for better comparability with data from the literature.

The categorical consideration of burnout, measured with MBI, allows a quick and rough estimate of the prevalence, which is sometimes necessary and helpful in a medical context. The difficulty here is that there are no standardized cut-off values for MBI. Therefore, after consultation with Prof. J. Glaser from the University of Innsbruck (oral communication in 2006 and 2016) we used the cutoff > 4.5 points (item mean) for the domain emotional exhaustion. According to the theory of Maslach et al. emotional exhaustion is considered the core dimension of burnout and the most obvious manifestation of this complex construct (35). Therefore, we focused solely on emotional exhaustion. This decision is also supported by the results of Wang et al. (36) and Weigl et al. (37). They report that emotional exhaustion in particular is significantly related to long working hours, high job effort and low reward (36) and that this dimension of burnout is particularly influenced by ERI with effects on the quality of care (37).

#### Effort-Reward-Imbalance

The effort-reward-imbalance questionnaire (ERI) measures effort and reward in the occupational context and relates them to each other (ERI ratio). Five items refer to effort, 11 items refer to reward. Higher effort is reflected in higher scores, ranging from 1 (does not apply) to 5 (does apply). The sum scores for effort range from 5 to 25. Lower reward is reflected in lower scores with the sum score from 11 to 55. To compute the ERI ratio, the effort score is divided by the reward score and then multiplied by a correction factor to correct the difference in the numbers of items of the two scales. Imbalance between effort and reward is present when the value is over 1 (29, 37). Overcommitment (OC) is recorded by 6 items ranging from 1 (low) to 4 (high) with sum scores from 6 to 24. A score > 16 indicates a high risk developing somatic or mental stress symptoms (13, 14, 29).

### Statistical Analysis

Statistical computations and data management were performed with the Statistical Package for the Social Sciences (SPSS), Version 24.0 and Stata 15.1 SE (Stata Corp. College Station, USA).

#### Bivariate Analysis

To identify differences between the cohorts in 2006 and 2016 with respect to occupational and personal factors as well as occupational stress and mental health, we first tested the significance of all categorical variables. To estimate the average unadjusted differences between the 2006 and 2016 surveys, we used Chi-square-tests. We tested continuous variables on skewness and kurtosis for normal (gaussian) distribution. If there was no normal distribution and different sample sizes, we used Mann-Whitney U Tests. Statistical significance was defined as *p* ≤ 0.05.

#### Statistical Matching

To compare the data sets of both years and to minimize the risk of bias through confounding covariates, we performed statistical matching. Propensity score matching was conducted prior to estimating models of change between the two cohorts (Cf. Table 2). A propensity score matching is appropriate for comparing exposed and non-exposed groups in observational data (38). In our study we used propensity scores from a number of matching variables that can be assumed to affect working time and job-related stress to compare similar findings on the outcome variable of interest. The matching variables were: sex (male vs. female), age (years), working full-time vs. part-time, working in hospital vs. private practice, leadership vs. subordinate position. Before calculating propensity scores we transferred missing data from all categorical variables into a missing category. This allowed us to integrate as much information as possible into the model (39).

**Table 2:** Average treatment (year) effect from observational data using logistic models after propensity-score matching (2016 vs. 2006), caliper 0.15.

***N* =1,797**	**ATE coef**.	**AI robust std. err**.	***z***	***P* > |*z*|**	**95% Conf. interval**	
Work hours (per week)	−0.21	0.68	−0.32	0.752	−1.54	1.11
Free weekends (per month)	0.33	0.06	5.9	0.00	0.22	0.44
ERI effort	−1.14	0.21	−5.48	0.00	−1.55	−0.73
ERI reward	3.60	0.38	9.38	0.00	2.85	4.36
ERI esteem	1.10	0.21	5.12	0.00	0.68	1.52
ERI job security	0.72	0.09	8.08	0.00	0.54	0.89
ERI job promotion	1.74	0.17	10.43	0.00	1.42	2.07
Effort-reward-ratio	−0.12	0.02	−8.2	0.00	−0.15	−0.10
Overcommitment	−0.79	0.21	−3.75	0.00	−1.21	−0.38
ERI categorial	−0.38	0.02	−4.64	0.00	−0.11	−0.05
OC categorial	−0.05	0.02	−2.08	0.04	−0.10	0.00
MBI-EE item scores	−0.35	0.05	−6.85	0.00	−0.45	−0.25
MBI-DP item scores	−0.16	0.04	−3.7	0.00	−0.24	−0.07
MBI-PA item scores	0.14	0.03	4.78	0.00	0.08	0.20
MBI-EE sum scores	−3.13	0.46	−6.85	0.00	−4.04	−2.24
MBI-DP sum scores	−0.79	0.21	−3.7	0.00	−1.20	−0.37
MBI-PA sum scores	0.10	0.21	4.78	0.00	0.58	1.39
MBI-EE categorial	−0.19	0.04	−4.78	0.00	−0.27	−0.11
MBI-DP categorial	−0.16	0.04	−4.33	0.00	−0.23	−0.08
MBI-PA categorial	0.05	0.02	2.66	0.01	0.01	0.09
BDI categorial	−0.08	0.02	−4.01	0.00	−0.12	−0.04
Work missed due overload	0.02	0.02	0.81	0.42	−0.02	0.05
Currently in psychotherapy	0.00	0.01	−0.04	0.97	−0.03	0.03
Previously in psychotherapy	−0.02	0.03	−0.83	0.41	−0.08	0.03
Depression in medical history	−0.06	0.03	−2.24	0.03	−0.12	−0.01
Suicide attempt	−0.01	0.01	−0.74	0.46	−0.02	0.01
Medication intake	0.08	0.03	3.15	0.00	0.03	0.13
Antidepressant intake	−0.01	0.01	−0.47	0.64	−0.03	0.02
Sedative intake	0.00	0.01	0.48	0.63	−0.02	0.03
Analgesic intake	0.04	0.02	2.6	0.01	0.01	0.07
Acute/chronic illness	0.09	0.03	3.4	0.00	0.04	0.15

*Matching variables were sex (men, women), age (years), full-time contract (no, yes, not reported); main occupation (private practice, hospital, not reported), working position (senior position, assistance position, not reported). AI, robust Abadie-Imbens standard errors with caliper of 0.15. Reading example: The average number of free weekends is significantly different between years indicating an average increase in free weekends of 0.33 per month*.

#### Correlation Analyses

We investigated potential correlations between socioeconomic and job factors and occupational stress and mental health by means of correlation analyses (Pearson's correlations) (31). We chose independent variables that we assumed to have an influence on the experience of stress: sex, working position, work hours, free weekends, having children, having a partner. Dependent variables were the scores of the rating scales (ERI, OC, MBI-EE). In the analysis of burnout, we only included emotional exhaustion, as this dimension is considered the core aspect of burnout and the main symptom (38). There is also evidence that emotional exhaustion is influenced by long working hours, high effort and low reward experience in the job, and the latter, in turn, influences the quality of care (31, 39). In the correlation analyses, we combined 2006 and 2016, as we wanted to know in general whether there were correlations between potential stress factors in professional and private life and mental health scores. The “not reported” categories were excluded from this analysis.

## Results

### Descriptive Data Analysis

In the decade studied, the proportion of participating females changed from 46.74% (2006) to 54.25% (2016). The mean age increased significantly by 3.9 years from 44.4 in 2006 to 48.3 years in 2016 (Cf. Table 1).

#### Private Life

In 2016 significantly fewer psychiatrists surveyed reported living in a relationship with a partner (2006: 80.34%; 2016: 76.00%) and significantly fewer psychiatrists reported having children in 2016 (62.12%) compared to 2006 (68.41%). The number of children living in the household of the participants at the time of the survey was significantly lower in 2006 (mean: 1.69) than in 2016 (mean: 1.19). (Cf. Table 1).

#### Occupational Life

In 2016 significantly fewer psychiatrists surveyed reported working full-time than in 2006 (reduction of around 12 percentage points, 2006: 84.65%; 2016: 72.12%). The number of those in a senior position was slightly higher in 2016 (57.63%) compared to 2006 (56.97%), while the percentage points of those in a subordinate position decreased by 4.3 percentage points. And in 2016 nearly 3 percentage points fewer psychiatrists surveyed reported working in private practice than in 2006 (28.12%), while the proportion of those working at the hospital remained virtually unchanged (2006: 66.10%; 2016: 65.3%) (Cf. Table 1).

### Changes in Working Hours, Medical History, and Health

#### Working Hours

The number of working hours per week was about 3 h higher among psychiatrists surveyed in 2006 than among psychiatrists surveyed in 2016. The number of weekends without being on duty was significantly higher in the 2016 sample (mean: 2.8) compared to the 2006 sample (mean: 2.35) (Cf. Table 1). Even the working hours of full-time employees were significantly higher in the 2006 sample (mean: 52.1) compared to 2016 (mean: 50.6). Hence, the reduction in working time in the surveyed decade cannot be solely attributed to more part-time work or a higher number of individuals likely to work part-time, such as women. (Cf. Table 1).

#### Health and Medical History

Despite the improvement in working time, we found no significant difference in the frequency of being on sick leave due to overload between the 2006 sample and 2016 sample (2006: 11.84%; 2016: 14.12%).

However, there was a significant increase of those who claimed to have an acute and/or chronic illness at the time the survey was taken [over 12 percentage points from 2006 (29.59%) to 2016 (41.12%)]. The number of participants taking medication was lower in the 2006 sample (24.67%) compared to the 2016 sample (37.62%). Especially the intake of analgesics has nearly doubled in the decade (2006: 6.62%; 2016: 11.00%). The groups did not differ significantly regarding the occurrence of depression in the medical history (2006: 41.83%; 2016: 38.00%). The number of those whose depression in the medical history has been diagnosed by a specialist, however, was significantly lower in the 2006 sample (32.7%) than in the 2016 sample (41.9%) (Cf. Table 1).

In 2016, significantly more of the psychiatrists surveyed were in ongoing psychotherapeutic treatment than in 2006 (6.62 vs. 4.81%). Meanwhile, the number of those who were previously in psychotherapeutic treatment remained stable. In 2016 fewer participants answered yes to the question about suicide attempts than in 2006 (*N* = 18 vs. *N* = 24). Since the number of cases is low, statements on significance must be interpreted critically.

#### Burnout Dimensions

**Figure 1:**
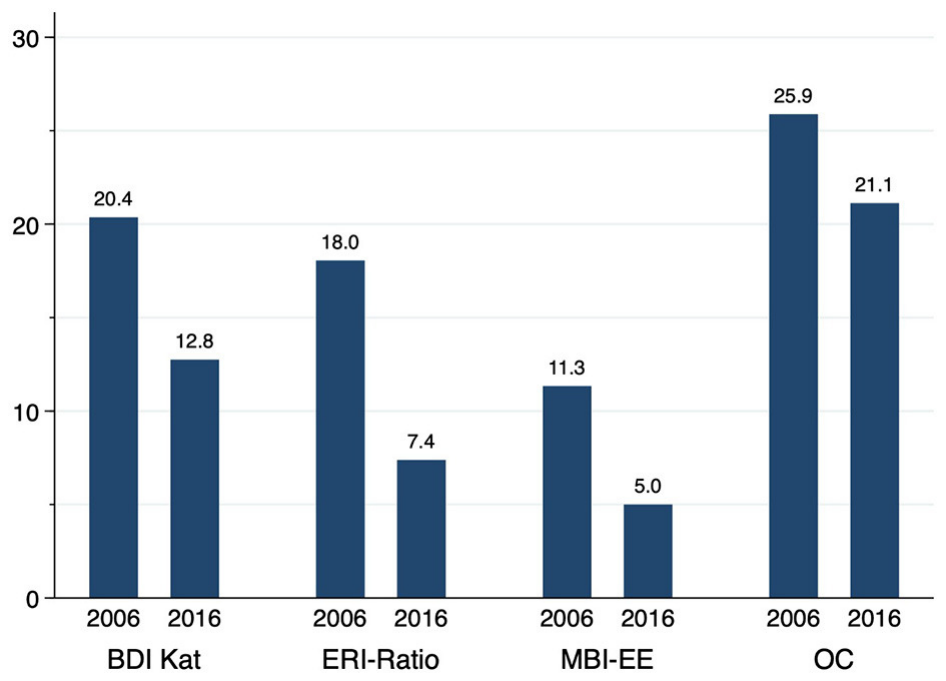
Percentage of surveyed psychiatrists 2006 and 2016 scoring high in the areas of clinically relevant depression (BDI > 10, BDI-II > 13), acute burnout risk (MBI-EE > 4.5), effort-reward-imbalance (ERI > 1), and overcommitment (OC > 16).

In all three dimensions of the burnout construct in the Maslach Burnout Inventory, a significant improvement was shown by item means and sum scores (Cf. Table 3A). Similarly, the dichotomous analysis of the data of the core dimension emotional exhaustion showed an improvement. In 2006 11.3% (*N* = 113) of the surveyed psychiatrists reached the 4.5 cutoff for the core dimension of emotional exhaustion. In 2016 it was 5.0% (*N* = 40) (*p* < 0.001; Cf. Figure 1).

**Table 3A:** Comparison of questionnaire scores of the study samples in 2006 and 2016.

**Questionnaire scores**	**2006**		**2016**			***D* 95% Conf. interval**
	**(*****N*** **=** **997)**		**(*****N*** **=** **800)**			
**MBI (item means)**	***M***	**SD**	***M***	**SD**	***p***	
Emotional exhaustion	3.31	0.952	2.93	0.91	<0.001	0.42 (0.32–0.51)
Depersonalization	2.28	0.768	2.09	0.738	<0.001	0.25 (0.16–0.34)
Personal accomplishment	4.8	0.524	4.95	0.513	<0.001	−0.29 (−0.38 to −0.19)
**MBI (sum scores)**						
Emotional exhaustion	29.8	8.57	26.3	8.19	<0.001	0.42 (0.32–0.51)
Depersonalization	11.4	3.84	10.5	3.69	<0.001	0.25 (0.16–0.34)
Personal accomplishment	33.6	3.66	34.7	3.59	<0.001	−0.29 (−0.38 to −0.19)
**ERI**						
Effort	14.7	3.77	13.4	3.66	<0.001	0.35 (0.26–0.45)
Reward	44.4	7.71	48.3	6.47	<0.001	−0.54 (−0.64 to −0.45)
Esteem	20.7	3.99	21.8	3.64	<0.001	−0.30 (−0.39 to −0.20)
Job security	8.25	1.89	9.06	1.52	<0.001	−0.47 (−0.56 to −0.37)
Job promotion	15.5	3.6	17.4	2.91	<0.001	−0.57 (−0.67 to −0.48)
Effort-reward ratio	0.774	0.334	0.633	0.247	<0.001	0.47 (0.38–0.57)
Overcommitment	14	3.65	13.2	3.6	<0.001	0.22 (0.13–0.31)

*P-Values from Mann-Whitney-U-Test. Effect size (31): Cohens D, D = 0.1 small effect, D = 0.3 medium effect, D = 0.5 large effect*.

#### Effort-Reward-Imbalance

The effort-reward ratio was significantly lower (by 10% points) in the 2016 sample compared to the 2006 sample (Cf. Table 3A). Values > 1, indicating an effort-reward-imbalance, were reached by 18% (*N* = 180) of the participants in 2006 and by 7.4%; (*N* = 59) in 2016 (*p* < 0.001). The number of those showing overcommitment was higher in the 2006 sample compared to the 2016 sample (2006: 25.9%, *N* = 258 vs. 2016: 21.1%, *N* = 169; *p* < 0.05) (Cf. Figure 1).

#### Depression

In 2016, 12.8% (*N* = 102) of the congress participants reached values in BDI-II that indicate depression. In 2006, even more (20.4%; *N* = 203) reached values indicating depression (BDI) *p* < 0.001. Since we used different versions of the BDI in 2006 and 2016, the sum scores are not directly comparable. We have shown the BDI sum scores in Table 3B.

**Table 3B:** Questionnaire scores BDI (2006) and BDI-II (2016).

**Questionnaire (sum scores)**	***N***	***M***	**SD**
BDI (2006)	997	6.76	5.69
BDI-II (2016)	800	6.36	6.16

*Due to the different versions of the BDI, no comparisons can be calculated*.

#### Correlations

The correlation analyses of socio-demographic, private and professional factors and effort-reward ratio, overcommitment and emotional exhaustion are shown in Table 4. We found a statistically significant positive correlation between working hours per week and values for ERI ratio (*R* = 0.2325), overcommitment (*R* = 0.1525) and emotional exhaustion (*R* = 0.2325). Similarly, we found a statistically significant negative correlation between weekends with on-call duty and values for ERI ratio (*R* = −0.2474), overcommitment (*R* = −0.2271) and emotional exhaustion (*R* = −0.2474). The private factor partnership showed a significant positive correlation with values for emotional exhaustion (*R* = −0.0715) and ERI ratio (*R* = −0.0715). Furthermore, a correlation was found between female gender and overcommitment (*R* = −0.1297).

**Table 4:** Results of the correlation analyses (combined 2006 and 2016).

	***N***	**ERI**	**OC**	**MBI-EE**
Gender (female vs. male)	1797	−0.0362	−0.1297^*^	−0.0362
Working position (senior vs. assistance)	1717	0.022	0.0146	0.0222
Work hours (per week)	1797	0.2325^*^	0.1525^*^	0.2325^*^
Free weekends (per month)	1797	−02474^*^	0.2271^*^	−0.2474^*^
Children	1082	0.0507	0.0145	0.0507
Partnership	1647	−0.0715^*^	0.0010	−0.0715^*^

OC, overcommitment; ERI, effort-reward ratio; MBI-EE, emotional exhaustion item scores. Correlation coefficient [Pearsons R; effect size: R = 0.1 small effect, R = 0.3 medium effect, R = 0.5 large effect; Cohen (31)],

* *p ≤ 0.05*.

## Discussion

In our study, we investigated changes in working hours, private and occupational stress factors, burnout and mental health among psychiatrists over a decade, during which a legislative change classified on-call duties as working time (22). Therefore, an initial expectation was that this legislative change would be reflected in a measurable reduction in working hours, as this would be a precondition to observe effects on burnout and mental health. The reduction of working hours was confirmed by our data. We were able to observe fewer weekly working hours and more free weekends (without on-call duty) in the sample in 2016 compared to the sample in 2006. It is plausible that this change was mainly due to the implementation of the Working Hours Act (22). To clarify whether the lower working time in 2016 may have been due to a higher proportion of part-time employees, we looked at the working hours of full-time employees separately, which would have been directly impacted by the legislation. The weekly working hours of full-time employees also decreased during the period under study. Another possible explanation of lower working hours may be a new attitude toward work and profession (40). However, we believe that a change in attitude cannot explain all observed effects on working hours. A change in attitude, for example, would have no influence on the number of duty weekends, as these have to be worked. Duty weekends were also lower in the 2016 sample.

The results on changes concerning effort-reward-imbalance, burnout and depressiveness are of key importance for our work, as the significant reduction in working time in the decade under study from 2006 to 2016 would be expected to be accompanied by decreases in these indices. According to our data, the 2016 sample of psychiatrists surveyed reported significantly fewer effort-reward-imbalances (7.4%) than the sample in 2006 (18.0%). Similar, overcommitment was reported to be significantly lower in the 2016 sample (21.1%) than in the 2006 sample (25.9%) (Cf. Figure 1). Correlation analyses also showed an association between working hours on the one hand and the extent of effort-reward-imbalance and overcommitment on the other.

As predicted by the effort-reward-imbalance model, our data also show an improvement in all three dimensions of MBI over the period studied, which indicates a lower risk of burnout. The difference in burnout risk between the 2006 sample and the 2016 sample becomes visible at a glance when looking at Figure 1, which shows that in the 2006 sample, 11.8% achieved the cut-off for burnout; in the 2016 sample it was 5%. These results would also be consistent with the burnout model according to Maslach and Jackson (35). Also here the improvements in the MBI correlated with reductions in working hours.

In the BDI and BDI-II, scores that are consistent with at least mild depression were recorded more frequently in the sample of 2006 than in the sample of 2016. This is consistent with the significantly decreased MBI scores of the participants from 2016 compared to the participants from 2006, as predicted by the effort-reward imbalance model (14, 29).

A companion study of intensive care physicians provided similar evidence that lower weekly working hours and more weekends off may have a positive influence on the occupational stress of physicians (41). As in the present sample, correlation analyses showed that more working hours per week and working days on weekends were associated with an increased effort-reward-imbalance and higher burnout scores in intensive care physicians. Regarding the number of hours worked per week, the surveyed intensive care physicians reported ~3 h less in 2016 than in 2006. Effort-reward-imbalance decreased from 23.9% in the 2006 sample to 14.7% in the 2016 sample and 8.2% were at risk of burnout in both years.

Notwithstanding the improvements in mental health indices from 2006 to 2016 in our sample, 2016 figures were still high relative to population levels. Even if our sample may have suffered from limited representativeness due to the recruitment method, occupational stress was similar (2006) or slightly lower (2016) than in the general population. There are data from 2008 suggesting an effort-reward-imbalance for almost 26% of the workforce (general German population) (42), which is higher than the figures from our 2006 sample (18%). In a survey of a German metropolitan transport company (*N* = 315) a few years earlier, 16.1% of the employees had an effort-reward-imbalance (43), which roughly corresponds to our figures from 2006 (18%).

In a survey of German physicians, 27.6% presented an effort-reward imbalance (44). This speaks for a lower effort-reward-imbalance in our sample (18% in 2016). Also, the proportion of intensive care physicians in our companion study with an effort-reward-imbalance was higher compared to the sample of psychiatrists (intensive care physicians 2016: 14.7%, psychiatrists 2016: 7.4%) (41). Our results speak against higher occupational stress levels among the psychiatrists we surveyed compared to data from other professional groups and physicians in other specialties which were roughly collected in the same period. Whether this tendency is also found in the overall population of psychiatrists and which factors have an influence on this should be the subject of further research.

The proportion of psychiatrists at burnout risk in our study sample is higher than the available values for the general population in Germany (burnout prevalence: 4.2%) (45). A smaller survey of physicians of various specialties in an East German medical association (“Ärztekammer”) (46), surveyed in 2012 yielded lower MBI sum scores (EE: 21.3, DP: 9.9, PE: 36.3) than we determined in our 2016 survey (EE: 26.3, DP: 10.5, PA: 34.7). In our companion study the proportion of intensive care physicians at burnout risk was 8.2% in 2016 and therefore higher than in our present study sample (5%). The lower levels of burnout among our sample of psychiatrists compared with intensive care physicians could be due to lower working hours in psychiatrists (2016: intensive care physicians: mean: 53.2; psychiatrists: mean:46.5).

Also, depression was higher in our sample than in the general population. According to our data, the at least mild depressiveness measured in the BDI-II, which was 12.8% in the 2016 sample, was above the point prevalence for a depression of 8.56% assumed for the general population, assessed with the BDI in a study from 2001 (47). More recent data on the prevalence of depression in Germany is provided by the GEDA 2014/2015-EHIS study, according to which the 12-month prevalence of self-reported diagnosed depression is 8.1% in the population (48).

These findings are also reflected in the results on medical history data, which show that the frequency of depressive episodes in the medical history was at a very high level (~40%) in both samples. This also applies to the number of attempted suicides (over 2%). Currently, in Germany the lifetime prevalence of depression is estimated in the ODIN study from 2001 at 11.6% (47), and the lifetime prevalence of attempted suicides is estimated at 1.7%, based on data from the early 2000 s (49).

What was surprising from our data was the low number of those who have had themselves examined by a specialist and of those undergoing psychotherapy (in both years). These findings could be a sign for stigma of mental illness and psychiatric help, even among mental health professionals (50–52). And maybe the higher rates of accepted professional help among participants in 2016 also contribute to fewer participants scoring at least mild depression in the BDI-II in 2016. This should be a prelude to further research, but also to the establishment of low-threshold support services specifically for physicians.

Psychiatry is a medical field of activity where the close proximity to the patient, the permanent handling of difficult emotions and stressful experiences (17–19) may require time without patient contacts to preserve healthy functioning. Our findings support the claim that a legislative intervention aiming at reducing working hours achieves the intended outcome of reducing occupational stress levels, emotional exhaustion, and depression.

Some limitations of this study need to be mentioned. First, we assumed that attendees of the DGPPN were a representative sample of German psychiatrists. However, physicians who may be particularly burdened or mentally ill do not attend conferences or participate in surveys. This would lead to an underestimation of the prevalence of burnout, depressiveness and effort-reward-imbalance in our study. A similar consideration applies to attendees who did not take part or dropped out of the survey. Regarding the return rate, it must be critically noted that although we know how many of the questionnaires we handed out were returned, we do not know how many congress participants we made aware of our study and how many of them decided to participate. Therefore, no reliable statement can be made about the response rate. Second, answering the questions of a self-assessment scale requires a certain amount of self-disclosure, which may lead to selective underreporting. However, we had little missing data in the emotionally more sensitive items in our survey, suggesting that underreporting was a minor problem. Nevertheless, we cannot rule out aggravated or trivialized answers to questions about the medical history and current symptoms. Another methodological criticism is that the effort-reward-imbalance questionnaire assesses occupational stress due to working time in one item, while we also assess working time and then relate it to occupational stress as measured by the effort-reward-imbalance-questionnaire.

## Conclusion

Our study is the first to document job-related and private stress factors and mental health in a large sample of psychiatrists after a statutory reduction in working hours. Our study shows positive changes in the period of a decade with regard to the time workload, occupational stress and indices of mental health. At the same time, the data show that the burden is high even after 9 years. The correlations between long working hours and effort-reward-imbalance and emotional exhaustion suggest that workplace-related preventive interventions in professions with high emotional stress could be promising. The results on overcommitment also give reason to think about individual prevention offers. Finally, it should be noted, that occurrence of burnout among the psychiatrists who participated in the survey is important, because burnout, in addition with the subjective suffering it involves, are also associated with a reduced work performance (7, 8).

## Data Availability Statement

The datasets generated in this article are not readily available because the participants were not informed that the data would be publicly available and therefore could not agree to this. Requests to access the datasets should be directed to Petra Beschoner, petra.beschoner@uni-ulm.de.

## Ethics Statement

The studies involving human participants were reviewed and approved by the Ethics Commission of the University of Ulm. The patients/participants provided their written informed consent was not required for this study in accordance with the national legislation and the institutional requirements.

## Author Contributions

MB, PB, CS-L, JW, and AB: conceptualization. RV, LJ-B, and MJ: methodology. MJ and MK: software. PB, AB, and MJ: validation. MJ, PB, and RV: formal analysis. AB, PB, and MB: investigation. MB, JW, CS-L, and MK: resources. MJ and PB: data curation. PB: writing—original draft preparation. LJ-B, AB, JW, and RV: writing—review and editing. MJ: visualization. JW and CS-L: supervision. AB and MK: project administration. MB and CS-L: funding acquisition. All authors: have read and agreed to the published version of the manuscript.

## Conflict of Interest

The authors declare that the research was conducted in the absence of any commercial or financial relationships that could be construed as a potential conflict of interest.

## References

[1] Weigl M Hornung S Petru R Glaser J Angerer P. Depressive symptoms in junior doctors: a follow-up study on work-related determinants. Int Arch Occup Environ Health. (2012) 85:559–70. 10.1007/s00420-011-0706-8 21956449

[2] Rotenstein LS Torre M Ramos MA Rosales RC Guille C Sen S. Prevalence of burnout among physicians: a systematic review. JAMA. (2018) 320:1131–50. 10.1001/jama.2018.12777 30326495 PMC6233645

[3] Liu L Chang Y Fu J Wang J Wang L. The mediating role of psychological capital on the association between occupational stress and depressive symptoms among Chinese physicians: a cross-sectional study. BMC Public Health. (2012) 12:219. 10.1186/1471-2458-12-219 22436106 PMC3410796

[4] Boxer PA Burnett C Swanson N. Suicide and occupation: a review of the literature. J Occup Environ Med. (1995) 37:442–52. 10.1097/00043764-199504000-00016 7670900

[5] Schernhammer ES Colditz GA. Suicide rates among physicians: a quantitative and gender assessment (meta-analysis). Am J Psychiatry. (2004) 161:2295–302. 10.1176/appi.ajp.161.12.2295 15569903

[6] Patel RS Bachu R Adikey A Malik M Shah M. Factors related to physician burnout and its consequences: a review. Behav Sci. (2018) 8:98. 10.3390/bs8110098 30366419 PMC6262585

[7] Dewa CS Loong D Bonato S Trojanowski L. The relationship between physician burnout and quality of healthcare in terms of safety and acceptability: a systematic review. BMJ Open. (2017) 7:e015141. 10.1136/bmjopen-2016-015141 28637730 PMC5734243

[8] Fahrenkopf AM Sectish TC Barger LK Sharek PJ Lewin D Chiang VW. Rates of medication errors among depressed and burnt out residents: prospective cohort study. BMJ. (2008) 336:488–91. 10.1136/bmj.39469.763218.BE 18258931 PMC2258399

[9] Tomioka K Morita N Saeki K Okamoto N Kurumatani N. Working hours, occupational stress and depression among physicians. Occup Med (Lond). (2011) 61:163–70. 10.1093/occmed/kqr004 21383384

[10] Martini S Arfken CL Balon R. Comparison of burnout among medical residents before and after the implementation of work hours limits. Acad Psychiatry. (2006) 30:352–5. 10.1176/appi.ap.30.4.352 16908615

[11] Gopal R Glasheen JJ Miyoshi TJ Prochazka AV. Burnout and internal medicine resident work-hour restrictions. Arch Intern Med. (2005) 165:2595–600. 10.1001/archinte.165.22.2595 16344416

[12] Shanafelt TD Mungo M Schmitgen J Storz KA Reeves D Hayes SN. Longitudinal study evaluating the association between physician burnout and changes in professional work effort. Mayo Clin Proc. (2016) 91:422–31. 10.1016/j.mayocp.2016.02.001 27046522

[13] Siegrist J Li J. Associations of extrinsic and intrinsic components of work stress with health: a systematic review of evidence on the effort-reward imbalance model. Int J Environ Res Public Health. (2016) 13:432. 10.3390/ijerph13040432 27104548 PMC4847094

[14] Siegrist J. Effort-reward imbalance model. In: Fink G, editor. Stress: Concepts, Cognition, Emotion, and Behavior. San Diego, CA: Academic Press (2016). p. 81–6.

[15] Rugulies R Aust B Madsen IE. Effort-reward imbalance at work and risk of depressive disorders. A systematic review and meta-analysis of prospective cohort studies. Scand J Work Environ Health. (2017) 43:294–306. 10.5271/sjweh.3632 28306759

[16] Dutheil F Aubert C Pereira B Dambrun M Moustafa F Mermillod M. Suicide among physicians and health-care workers: a systematic review and meta-analysis. PLoS ONE. (2019) 14:e0226361. 10.1371/journal.pone.0226361 31830138 PMC6907772

[17] Ruskin R Sakinofsky I Bagby RM Dickens S Sousa G. Impact of patient suicide on psychiatrists and psychiatric trainees. Acad Psychiatry. (2004) 28:104–10. 10.1176/appi.ap.28.2.104 15298861

[18] Daniels J. Sekundäre Traumatisierung. Psychotherapeut. (2008) 53:100–7. 10.1007/s00278-008-0585-y

[19] Fothergill A Edwards D Burnard P. Stress, burnout, coping and stress management in psychiatrists: findings from a systematic review. Int J Soc Psychiatry. (2004) 50:54–65. 10.1177/0020764004040953 15143847

[20] Rosta J. Hospital doctors' working hours in Germany-preliminary data from a national survey in autumn 2006. Deutsches Arzteblatt-Koln. (2007) 104:2067. Available online at: https://www.aerzteblatt.de/int/archive/article/58149/Hospital-Doctors-Working-Hours-in-Germany-Preliminary-Data-from-a-National-Survey-in-br-Autumn-2006 (accessed March 22, 2021).

[21] Hautzinger M Bailer M Worall H Keller F. Beck-Depressions-Inventar (BDI): Bearbeitung der deutschen Ausgabe. Göttingen: Germany Verlag Hans Huber (1994).

[22] Bernd G. Arbeitsrecht: Bereitschaftsdienst ist Arbeitszeit. Dtsch Arztebl Int. (2013) 110:2. Available online at: https://www.aerzteblatt.de/archiv/146754/Arbeitsrecht-Bereitschaftsdienst-ist-Arbeitszeit (accessed March 22, 2021).

[23] Reppel S. Überlange Arbeitszeiten im Krankenhaus: Wer haftet bei Fehlern? Dtsch Arztebl Int. (2014) 111:2. Available online at: https://www.aerzteblatt.de/archiv/154608/Ueberlange-Arbeitszeiten-im-Krankenhaus-Wer-haftet-bei-Fehlern (accessed March 22, 2021).

[24] Wittmann H. DGPPN Kongress 2010 in Berlin. Available online at: http://blog.klett-cotta.de/news-medien/dgppn-kongress-2010-in-berlin (accessed March 19, 2021).

[25] Sax LJ Gilmartin SK Bryant AN. Assessing response rates and nonresponse bias in web and paper surveys. Res Higher Educ. (2003) 44:409–32. 10.1023/A:1024232915870

[26] Beck AT Ward CH Mendelson M Mock J Erbaugh J. An inventory for measuring depression. Arch Gen Psychiatry. (1961) 4:561–71. 10.1001/archpsyc.1961.01710120031004 13688369

[27] Hautzinger M Keller F Kühner C. BDI-II Beck Depressions-Inventar—Revision. Frankfurt am Main: Pearson Assessment & Information GmbH (2009).

[28] Maslach C Jackson SE Leiter MP. Maslach Burnout Inventory. Palo Alto, CA: Manual. Consulting Psychologists Press (1996).

[29] Siegrist J Starke D Chandola T Godin I Marmot M Niedhammer I. The measurement of effort-reward imbalance at work: European comparisons. Soc Sci Med. (2004) 58:1483–99. 10.1016/S0277-9536(03)00351-4 14759692

[30] Kühner C Bürger C Keller F Hautzinger M. Reliabilität und Validität des revidierten Beck-Depressionsinventars (BDI-II). Befunde aus deutschsprachigen Stichproben. Nervenarzt. (2007) 78:651–6. 10.1007/s00115-006-2098-7 16832698

[31] Cohen J. Statistical Power Analysis for the Behavioral Sciences. 2nd ed. Hillsdale, NJ: Lawrence Erlbaum Associates (1988).

[32] Alexandrowicz RW Fritzsche S Keller F. Die Anwendbarkeit des BDI-II in klinischen und nichtklinischen Populationen aus psychometrischer Sicht. Eine vergleichende Analyse mit dem Rasch-Modell. Neuropsychiatrie. (2014) 28:63–73. 10.1007/s40211-014-0104-z 24848375

[33] Büssing A Perrar KM. Die Messung von Burnout. Untersuchung einer deutschen Fassung des Maslach Burnout Inventory (MBI-D). Diagnostica. (1992) 38:328–53. 8004334

[34] Burisch M. Das Burnout-Syndrom. Theorie der inneren Erschöpfung. 5th ed. Heidelberg: Springer (2010).

[35] Maslach C Schaufeli WB Leiter MP. Job burnout. Annu Rev Psychol. (2001) 52:397–422. 10.1146/annurev.psych.52.1.397 11148311

[36] Wang Z Xie Z Dai J Zhang L Huang Y Chen B. Physician burnout and its associated factors: a cross-sectional study in Shanghai. J Occup Health. (2014) 56:73–83. 10.1539/joh.13-0108-OA 24430838

[37] Weigl M Schneider A Hoffmann F Angerer P. Work stress, burnout, and perceived quality of care: a cross-sectional study among hospital pediatricians. Eur J Pediatr. (2015) 174:1237–46. 10.1007/s00431-015-2529-1 25846697

[38] Rosenbaum PR Rubin DB. The central role of the propensity score in observational studies for causal effects. Biometrika. (1983) 70:41–55. 10.1093/biomet/70.1.41

[39] Gould W. Ladder-of-powers variable transformation. Stata Technical Bulletin. (1992) 1:14–5.

[40] Kasch R Engelhardt M Förch M Merk H Walcher F Fröhlich S. Physician shortage: how to prevent generation Y from staying away—results of a nationwide survey. Zentralbl Chir. (2016) 141:190–6. 10.1055/s-0035-1557857 26394048

[41] Beschoner P von Wietersheim J Jarczok MN Braun M Schönfeldt-Lecuona C Jerg-Bretzke L. Changes in working conditions and mental health among intensive care physicians across a decade. Front Psychiatry. (2020) 11:145. 10.3389/fpsyt.2020.00145 32296349 PMC7136524

[42] Bethge M Radoschewski FM Gutenbrunner C. Effort-reward imbalance and work ability: cross-sectional and longitudinal findings from the Second German Sociomedical Panel of Employees. BMC Public Health. (2012) 12:875. 10.1186/1471-2458-12-875 23067110 PMC3505747

[43] Larisch M Joksimovic L von dem Knesebeck O Starke D Siegrist J. Effort-reward imbalance at work and depressive symptoms–a cross-sectional investigation of middle-aged employees. Psychother Psychosom Med Psychol. (2003) 53:223–8. 10.1055/s-2003-38867 12709890

[44] Voltmer E Rosta J Siegrist J Aasland OG. Job stress and job satisfaction of physicians in private practice: comparison of German and Norwegian physicians. Int Arch Occup Environ Health. (2012) 85:819–28. 10.1007/s00420-011-0725-5 22160090

[45] Kurth B-M. Erste Ergebnisse aus der “Studie zur Gesundheit Erwachsener in Deutschland” (DEGS). Bundesgesundheitsbl. (2012) 55:980–90. 10.1007/s00103-011-1504-529138901

[46] Pantenburg B Luppa M König HH Riedel-Heller SG. Burnout among young physicians and its association with physicians' wishes to leave: results of a survey in Saxony, Germany. J Occup Med Toxicol. (2016) 11:2. 10.1186/s12995-016-0091-z 26807138 PMC4724157

[47] Ayuso-Mateos JL Vázquez-Barquero JL Dowrick C Lehtinen V Dalgard OS Casey P. Depressive disorders in Europe: prevalence figures from the ODIN study. Br J Psychiatry. (2001) 179:308–16. 10.1192/bjp.179.4.308 11581110

[48] Thom J Kuhnert R Born S Hapke U. 12-Monats-Prävalenz der selbstberichteten ärztlich diagnostizierten Depression in Deutschland. J Health Monit. (2017) 2. 10.17886/RKI-GBE-2017-057 PMC10165904 37168947

[49] Teismann T Dorrmann W. Suizidalität - Risikoabschätzung und Krisenintervention. Psychotherapeut. (2013) 58:297–311. 10.1007/s00278-013-0984-6

[50] Knaak S Mantler E Szeto A. Mental illness-related stigma in healthcare: barriers to access and care and evidence-based solutions. Healthc Manage Forum. (2017) 30:111–6. 10.1177/0840470416679413 28929889 PMC5347358

[51] Tillett R. The patient within—psychopathology in the helping professions. Adv Psychiatr Treat. (2003) 9:272–9. 10.1192/apt.9.4.272

[52] Peris TS Teachman BA Nosek BA. Implicit and explicit stigma of mental illness: links to clinical care. J Nerv Ment Dis. (2008) 196:752–60. 10.1097/NMD.0b013e3181879dfd 18852619

